# Beverage Intake Patterns in a National Sample of Polish Adolescents: PLACE-19 Study

**DOI:** 10.3390/nu18091384

**Published:** 2026-04-28

**Authors:** Dominika Głąbska, Dominika Skolmowska, Dominika Guzek

**Affiliations:** 1 Department of Dietetics, Institute of Human Nutrition Sciences, Warsaw University of Life Sciences (SGGW-WULS), 159C Nowoursynowska Street, 02-776 Warsaw, Poland; dominika_glabska@sggw.edu.pl (D.G.); dominika_skolmowska@sggw.edu.pl (D.S.); 2 Department of Food Market and Consumer Research, Institute of Human Nutrition Sciences, Warsaw University of Life Sciences (SGGW-WULS), 159C Nowoursynowska Street, 02-776 Warsaw, Poland

**Keywords:** beverage, drink, sugar-sweetened beverages (SSBs), carbonated soft drinks (CSDs), adolescents, PLACE-19 Study

## Abstract

Background/Objectives: Insufficient hydration and high consumption of sugar-sweetened beverages (SSBs) is a significant public health challenge among adolescents. The aim of the presented study was to assess the beverage intake, in terms of quantity and choices, in a national-based population of Polish adolescents within the PLACE-19 Study. Methods: Within the PLACE-19 Study, the population of *n* = 1027 secondary school adolescents (age 15–20 years) was recruited based on a quota sampling of Polish secondary schools and *n* = 984 individuals (*n* = 674 female, *n* = 310 male) were included in the analysis. The study assessed beverages intake using the National Youth Physical Activity and Nutrition Study (NYPANS) survey questionnaire and data were gathered using the Computer-Assisted Web Interview (CAWI) technique. The beverages were assessed in the following groups: water, milk beverages, 100% fruit juices, coffee/tea, regular carbonated soft drinks (CSDs), diet CSDs, other sugar-sweetened beverages (SSBs); additionally, total SSBs were summarized. The total beverage intake was compared with 80% of the Adequate Intake (AI) level. Results: The highest intake was observed for water (250 mL per day), and tea beverages (178.1 mL per day). However, in combining SSBs, the total daily intake for this group was 394.4 mL per day. The highest share of the daily intake of beverages was attributed to water intake (23.81%), and tea beverages (11.45%), followed by 100% fruit juices (7.14%), fruit drinks (6.67%), and milk (6.45%); however, the intake of total combined SSBs was even higher than for water (32.47%). Male individuals were characterized by a higher share of milk beverages, regular CSDs, diet CSDs, and other SSBs than female ones, while female individuals were characterized by a higher share of water, and coffee/tea in their total daily beverage intake than male ones (*p* < 0.001). Younger individuals were characterized by a higher share of other SSBs in their total daily beverage intake than older ones (*p* = 0.034). For the vast majority of the studied group, the inadequate intake of beverages was observed, as over 70% of the studied sub-groups did not meet the AI value. Conclusions: The inadequate beverage intake in a population of adolescents may be a problem, especially considering high intake of SSBs, and relatively low intake of water. Especially in male and younger individuals, due to a higher intake of SSBs, than for female and older individuals, education is necessary to promote adequate intake and choices of beverages. Further research is needed in the area of beverage consumption to understand the determinants of beverage consumption and develop opportunities to improve it.

## 1. Introduction

Hydration is a complex phenomenon, which may be perceived as a state (the volume of total body water at a given time) or a process (the volume of water added to and removed from the total body water pool each day) [1]. Independently from a definition, it is associated with total body water percentage, which from the age of 3 years generally ranges from about 40% to over 60%, while decreasing with age, and body mass, as well as being higher in male than in female individuals [2].

Recently, a growing evidence emphasizes the role of adequate hydration for maintaining general health, including proper cognitive function, reduced risk of kidney stones, and weight management [3]. In spite of the fact that dehydration may be easily prevented, low-drinkers are commonly not noticed and not educated [4]. Among the population groups most prone to dehydration there are mainly infants and young children [5], as well as older adults, especially those with chronic diseases and in institutionalized settings [6]. However, the school-age population of students is also indicated as one that should also be considered, as the environment and age-mates do not promote adequate beverage intake and choices, due to low availability, low health literacy, and specific social conditions that may encourage students to make unhealthy choices of sugar-sweetened beverages (SSBs) [7].

While assessing positions by the World Health Organization (WHO) associated with beverage intake in children and adolescents, two major global public health challenges may be indicated: inadequate consumption of beverages leading to suboptimal hydration [8] and improper choices of beverages associated with excessive intake of SSBs [9].

The recent systematic review and meta-analysis by Papaoikonomou et al. [7], assessing hydration status of children and adolescents, indicated that the majority of studies report common dehydration in this age group, which is associated with inadequate water intake. While assessing water supply, various approaches are applied, based on various definitions, which either include or exclude water derived from foods other than beverages, but commonly, the term ‘beverages intake’ is used [10], based on definition of Popkin et al. [11], including water and all other fluids consumed as a drink, but excluding liquid meal replacement products, soups and other water-containing foods.

The recommendations of beverages intake by various authorities may differ based on definitions, but the common conclusion indicates than the beverages intake in adolescents is not adequate [12]. According to the opinion of the European Food Safety Authority (EFSA) Panel on Dietetic Products, Nutrition, and Allergies (NDA) [13], adolescents aged ≥14 years, with respect to water intake, are considered as adults, and the adequate intake (AI) for them is indicated as 2.0 L for female and 2.5 L for male individuals. According to the Institute of Medicine (IOM) Food and Nutrition Board [14], the AI for adolescents differs from this for adults and for the age of 14–18 years; it is defined as 2.3 L of total water, including 1.8 L of beverages for female and 3.3 L of total water, including 2.6 L of beverages for male individuals (while for the age group of 19–30 years, it is defined as 2.7 L and 3.7 L of total water, including 2.2 L and 3.0 L of beverages, for female and male individuals, respectively). According to the Polish National Institute of Public Health (PZH-NIH) [15], the AI for adolescents depends on age, and for individuals of 13–15 years, it is formulated as 1.95 L and 2.35 L for female and male adolescents, respectively, but for individuals aged ≥16 years it is the same as for adults—2.0 L and 2.5 L, for female and male adults, respectively.

For the total daily consumption of beverages, within the Healthy Lifestyle in Europe by Nutrition in Adolescence Cross Sectional Study (HELENA-CSS) [16], conducted in a population of adolescents aged 12.5–17.5 years from eight countries and published in 2011, it was observed that the beverage consumed by the largest percent of adolescents was water, followed by SSBs, fruit juice, and sweetened milk, while the mean beverage consumption was 1.3 L for girls and 1.6 L for boys. The Polish adolescents were not included to the study, but the study by Kostecka et al. [17] presenting data for 2018 and 2023 indicated a little bit higher intake for adolescents from voivodeships in central-eastern Poland, as in 2018 the mean beverage consumption was 1.6 L for girls and 1.8 L for boys, and in 2023 it was 1.7 L for girls and 2.0 L for boys. However, the intake was diverse in the studied group and still inadequate, as independently from the year, in a population of Polish adolescents only about 20% met daily beverage intake requirements [17].

For the choices of beverages, the study by Guelinckx et al. [18] published in 2015 and presenting beverages intake by children and adolescents from 13 countries, indicated that water was the major beverage consumed by adolescents, but its intake varied between countries, while the category defined within the study as regular soft beverages was characterized by the significant share in daily beverage intake. Moreover, further analysis of the database of SSBs intake for children and adolescents aged 3–19 years from 185 countries revealed that it increased by 23% from 1990 to 2018 [19]. In the referred study by Guelinckx et al. [18], compared with the other countries, for Poland, the lowest intake of water and the highest intake of hot beverages was observed, and the category of regular soft beverages was the second highest contributor to the total fluid intake after hot beverages.

Based on the presented data, it may be indicated that for adolescents in Poland, similarly as for the other countries, there are two problems related to beverage consumption, which should be assessed combined—inadequate intake and inappropriate choices. However, the problem of the choice of beverages may be for Poland even more serious than for the other countries included for international comparisons, due to the lowest intake of water [18] and a high intake of hot beverages, which include mainly hot tea, declared by Polish adolescents to be sweetened almost twice as often as unsweetened [20].

In Poland, no comprehensive analysis of beverage intake in the adolescent population, covering nationwide sample and various beverage categories, had been conducted so far; therefore, there was need to fill this gap. Taking this into account, the aim of the presented study was to assess the beverage intake, both in terms of quantity and choices, in a national-based population of Polish adolescents within the PLACE-19 Study.

## 2. Materials and Methods

### 2.1. General Information

The presented analysis was conducted based on the data gathered within the 4th phase of the PLACE-19 Study (Polish Adolescents’ COVID-19 Experience Study), taking place from May to June, 2021. It was the period when in Poland there was no longer lockdown, when the restrictions were relaxed, and school education was conducted as before the COVID-19 pandemic [21]. While the first phase of the PLACE-19 Study focused on hygienic and personal protective behaviors [22], the second phase examined general nutritional behaviors [23], the third phase addressed psychological aspects of food consumption [24], the fourth phase assessed detailed nutritional behaviors, including beverage consumption. The fourth phase was designed to fill the information gap regarding the quantitative and qualitative patterns of fluid intake after the prolonged period of the pandemic.

The PLACE-19 Study was conducted and supervised by the Institute of Human Nutrition Sciences, Warsaw University of Life Sciences (WULS-SGGW), based on the agreement obtained from the Ethics Committee of the Central Clinical Hospital of the Ministry of Interior and Administration in Warsaw (No. 2/2021). All the study procedures were planned and conducted on the basis of the guidelines of the Declaration of Helsinki, while participants of the study, and their parents/legal guardians, expressed their informed consent for participation in the PLACE-19 Study and all the study procedures.

### 2.2. Population Studied Within the PLACE-19 Study

The PLACE-19 Study was conducted based on the recruitment of secondary school students, where specific secondary schools were chosen within a quota sampling procedure. Such an approach was applied due to high Net Enrollment Rate (NER) in Poland for secondary school education at 89.38% for December 2019 (calculated on the basis of data from the Central Statistical Office (CSO) in Poland [25]).

Both sampling of secondary schools and recruitment of students were conducted separately for each phase of the PLACE-19 Study. Poland was administratively divided into 16 basic units (voivodeships), and further division of the voivodeships into following units (counties) was the basis for a quota sampling procedure, where the national register of the secondary schools was used to select schools. From each voivodeship, a total of 5 counties were randomly selected (resulting in total number of 16 × 5 = 80 counties), and from each county, a total of 5 secondary schools were randomly selected (resulting in total number of 80 × 5 = 400 schools).

After random choice of secondary schools from all the voivodeships of Poland, the headmaster of each of school was contacted and each school was invited to participate in the study. If headmaster agreed for the school participation in the PLACE-19 Study, the students from the school and their parents/legal guardians were informed, and their informed consents were gathered. After providing the informed consents, the students received the link to an electronic version of the study questionnaire and the responses were gathered using the technique of the Computer-Assisted Web Interview (CAWI).

For the 4th phase of the PLACE-19 Study, the following inclusion criteria were applied:

– Adolescents aged 15–20 years;
– Students from the secondary schools randomly chosen within sampling for the 4th phase of the PLACE-19 Study;
– Informed consent provided;
– For minor adolescents: informed consent of parents/legal guardians provided.

For the 4th phase of the PLACE-19 Study, the following exclusion criteria were applied:

– Participation in any previous phase of the PLACE-19 Study;
– Any missing or unreliable data within the questionnaire used for the analysis.

On the basis of the inclusion criteria, *n* = 1027 individuals were included to the 4th phase of the PLACE-19 Study, but afterwards, based on the exclusion criteria, *n* = 43 individuals were excluded: *n* = 9 due to missing data and *n* = 34 due to unreliable data. In order to define provided answers as unreliable, the mean total weekly beverage consumption was calculated, and the cut-off applied within analysis of the data according to the International Food Policy Study (IFPS) and the Canada Food Study (CFS) [26]; namely, responses summing up to >36 L per week were defined as nonsensical/implausible data. At the same time, responses denying consumption of any beverage were also defined as nonsensical/implausible. After excluding, the final number of responses analyzed within the study of beverage consumption were *n* = 984.

The sample size was calculated assuming a population of Polish adolescents aged 10–16 years (*n* = 2,726,492, based on the data from the Central Statistical Office in Poland [27]), a 95% confidence level, and a 5% margin of error. Assuming a proportion of 50%, the minimum required sample size was estimated at 384 respondents. Thus, the sample recruited within PLACE-19 Study, as well as the sample size of 984 respondents was interpreted as sufficient.

### 2.3. Questionnaire Applied Within the PLACE-19 Study

In order to assess the beverage intake, the National Youth Physical Activity and Nutrition Study (NYPANS) survey questionnaire was applied [28]. This questionnaire is based on a simple question about an intake of beverages during the past 7 days, while respondent is asked about a number of servings. In the original NYPANS survey questionnaire, the unified serving size of 237 mL was applied, except from 100% fruit juice with the serving size of 118 mL. In the questionnaire applied in the presented study, the serving size was changed into 250 mL for all beverages, as in Poland it is indicated as a standard serving size for non-alcoholic beverages [29]. The other change concerned the responses, as the original NYPANS survey questionnaire contained single-choice questions about the number of servings, which were changed into open-ended questions about the number of servings allowing both integers and decimals. This was decided, as in Poland, there are Food Frequency Questionnaires (FFQs) with open-ended questions about the number of servings applied [30,31]. Moreover, it was intended to not limit the declared frequency to the most typical, as the wrong frequency is indicated by the Food and Agriculture Organization of the United Nations (FAO) among the main sources of errors for the FFQ method [32].

In the original NYPANS survey questionnaire the standard beverage groups are applied, based on the Nutrition Data System for Research (NSDR), developed by the Nutrition Coordinating Center at the University of Minnesota [33], as follows: (1) water, (2) milk, (3) 100% fruit juices, (4) regular carbonated soft drinks (CSDs), (5) diet CSDs, (6) other SSBs, (7) coffee, coffee drinks, tea, with additional questions about: (8) isotonic drinks, and (9) energy drinks [29]. In the conducted study the same categories were applied, with the isotonic drinks, and energy drinks kept as important within the Polish beverage market and also in a group of adolescents [34]. At the same time, the different sub-groups were presented with examples within each category, to be applicable for a Polish beverage market, while for a milk the plant-based milk alternatives were also allowed. Moreover, within the categories of regular CSDs and diet CSDs, two separate questions were asked—about cola drinks and non-cola drinks. At the same time, within the category of coffee, coffee drinks and tea, two separate questions were asked—about coffee beverages and tea beverages (which were defined as those prepared at home or purchased in restaurants, to be distinguished from those purchased in shops). Last but not least, within the category of other SSBs, four separate questions were formulated in the presented study—about fruit drinks (different than 100% juices), flavored milk, bottled tea-based and coffee-based beverages (which were defined as those purchased in shops), and lemonade/flavored water. The additional questions about sub-groups were associated with some differences of the beverage market in Poland, including the increasing role of flavored waters, where in Poland the majority of them contain sugar [35], perceiving cola and non-cola drinks differently on the Polish market [36], and a high consumption of hot beverages [18].

Additionally, in the questionnaire there were questions allowing assessment of the inclusion and exclusion criteria (age, secondary school, participation in any previous phase of the PLACE-19 Study), and questions about the baseline characteristics of the participants (gender, weight, height).

### 2.4. Data Analysis Within the PLACE-19 Study

While assessing the obtained data, the beverages were analyzed according to a same groups as in the original NYPANS survey [28], based on the NSDR beverage groups [33], as follows: (1) water, (2) milk beverages (milk and flavored milk combined), (3) 100% fruit juices, (4) coffee/tea (coffee-beverages, tea-beverages, and bottled tea-based and coffee-based beverages combined), (5) regular CSDs (cola and non-cola CSDs combined), (6) diet CSDs (cola and non-cola diet CSDs combined), (7) other SSBs (fruit drinks, isotonic drinks, energy drinks and lemonade/flavored water combined). Additionally, the separate analysis was conducted for a group of SSBs combined (cola CSDs, non-cola CSDs, fruit drinks, isotonic drinks, energy drinks, lemonade/flavored water, flavored milk, bottled tea-based and coffee-based beverages).

Due to the fact that the original NYPANS survey questionnaire does not contain a question about specific quantity of servings, it does not allow the accurate assessment of the amount of beverages consumed [33]. However, the questionnaire applied in the presented study contained open-ended questions about number of servings, so such assessment was possible, but still, it must be taken into account, that FFQs for water intake are not the most accurate methods [37], and in general FFQs tend to overestimate [38]. Based on the applied questionnaire, the total daily beverage intake was calculated as a sum of intakes of specific beverages, and it was compared with the reference value in order to indicate the number of respondents characterized by adequate intake. However, as only intake of water from beverages and not from the other dishes was assessed, the AI values were not applicable, so the same approach as applied by other authors was chosen and 80% of the AI value was used for comparison [39], as it is assumed that 20% of water is derived from food other than beverages [13]. For calculation, the AI values by the following authorities were applied independently: (1) EFSA—adolescents aged ≥14 years: 2.0 L for female and 2.5 L for male individuals [13]; (2) IOM—adolescents aged 14–18 years: 2.3 L for female and 3.3 L for male individuals; aged ≥19 years: 2.7 L for female and 3.7 L for male individuals [14]; and (3) PZH-NIH—adolescents aged 13–15 years: 1.95 L for female and 2.35 L for male individuals; aged ≥16 years: 2.0 L for female and 2.5 L for male individuals [15].

The obtained results were compared between sub-groups, based on: (1) gender—female and male individuals, (2) age—15–17 years and 18–20 years, (3) body mass—underweight/normal body weight and excessive body weight. The body mass was assessed based on self-reported data on weight, height and the calculated Body Mass Index (BMI), while depending on age group, a different approach was applied to interpret the BMI value. For adult individuals (age ≥ 18 years), the standard BMI cut-offs were applied, as follows: underweight (<18.5 kg/m^2^), normal body weight (18.5–25.0 kg/m^2^), overweight (25.0–30.0 kg/m^2^) and obesity (>30.0 kg/m^2^) [40]. For minor individuals (age < 18 years), the standard BMI percentile cut-offs by WHO were applied, as follows: underweight (<5th percentile), normal body weight (5th–85th percentile), overweight (85th–95th percentile) and obesity (>95th percentile) [41], where for percentile value, the Polish gender- and age-specific growth reference values were used [42], accompanied by the Polish OLAF growth charts [43].

### 2.5. Statistical Analysis

The distribution was assessed for its normality using the Shapiro–Wilk test. While comparing subgroups, the Mann–Whitney U test was used (due to non-parametric distribution) with Bonferroni correction (in order to reduce the risk of false positive results), and for categorical variables the chi^2^ test was used with Yates’s correction for continuity. To indicate statistical significance a standard cut-off of *p* ≤ 0.05 was applied. The statistical analysis was conducted while using the Statistica 8.0 (Statsoft Inc., Tulsa, OK, USA) and the Jamovi 2.6.44 (The Jamovi Project, Sydney, Australia).

## 3. Results

The general characteristics of the group of adolescents studied within the PLACE-19 Study are presented in Table 1. Within the studied group of *n* = 984 individuals, there were *n* = 674 female ones and *n* = 310 male ones, while at the same time *n* = 674 were individuals aged <18 years. In the studied group, the majority of respondents were of normal body weight (70.8%), as well as there were also respondents with excessive body mass (22.2%), and who were underweight (6.9%). While comparing body mass of female and male individuals, there was a gender-dependent difference (*p* = 0.0360), and a higher share of female individuals were of underweight (8.0% vs. 4.5%, *p* = 0.0430).

The daily intake of beverages, in product sub-groups, assessed in a group of adolescents studied within the PLACE-19 Study, is presented in Table 2. The highest intake was observed for water (median of 1 serving of 250 mL per day), and tea beverages (median of 0.43 serving amounting 178.1 mL per day). However, while combining SSBs, the median of total daily intake for this group was 1.14 servings, amounting 394.4 mL per day. Similarly, the highest intake in the studied group (Q4) was observed for SSBs, amounting 3035 mL, while for none of the other product sub-groups the Q4 value exceeded 1500 mL. In the assessment within the survey questionnaire groups, the only groups characterized by the consumption of at least one serving (250 mL) per day were: water and coffee/tea (median of 250 mL for both groups).

The share of daily intake of beverages, in product sub-groups, assessed in a group of adolescents studied within the PLACE-19 Study, is presented in Table 3. The highest share of daily intake of beverages was attributed to water intake (median of 23.81%), and tea beverages (11.45%), followed by 100% fruit juices (7.14%), fruit drinks (6.67%), and milk (6.45%); however, when calculated the total SSBs combined share was even higher than for water (32.47%). The highest share of daily intake of beverages summed up in the groups, was attributed to water intake (median of 23.81%), coffee/tea (24.11%), and the group of other SSBs (18.30%).

The daily intake of beverages, in product sub-groups, assessed in a group of adolescents studied within the PLACE-19 Study, stratified by gender, is presented in Table 4. When comparing beverages intake, there were statistically significant gender-dependent differences for multiple beverage sub-groups. Male individuals were characterized by a higher intake of milk (*p* = 0.003), regular cola CSDs (*p* < 0.001), regular non-cola CSDs (*p* < 0.001), diet cola CSDs (*p* = 0.003), diet non-cola CSDs (*p* = 0.002), flavored milk (*p* < 0.001), lemonade/flavored water (*p* = 0.002), isotonic drinks (*p* < 0.001), and energy drinks, than female individuals (*p* = 0.002), as well as a similar difference was noted for total SSBs (*p* < 0.001).

**Figure 1:**
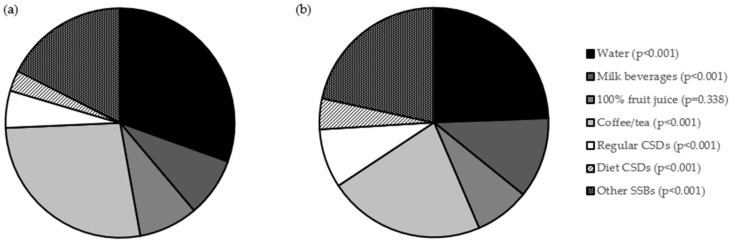
Share of daily intake of beverages, in product groups based on the survey questionnaire, assessed in a group of adolescents studied within the PLACE-19 Study (*n* = 984), stratified by gender, for female (**a**) and male individuals (**b**). Groups according to National Youth Physical Activity and Nutrition Study (NYPANS) survey [27], based on the Nutrition Data System for Research (NSDR), beverage groups [32]; *p*-Values for Mann–Whitney U test; CSDs—carbonated soft drinks; and SSBs—sugar-sweetened beverages.

The share of the daily intake of beverages, in product groups based on the survey questionnaire, assessed in a group of adolescents studied within the PLACE-19 Study, stratified by gender, is presented in Figure 1. Similarly as for beverages intake, for share of daily intake, for multiple beverages there were statistically significant gender-dependent differences. While compared with female individuals, male ones were characterized by a higher share of milk beverages (median of 9.45% vs. 7.14%; *p* < 0.001), regular CSDs (median of 6.56% vs. 3.03%; *p* < 0.001), diet CSDs (*p* < 0.001), and other SSBs in their total daily beverage intake (median of 21.43% vs. 17.02%; *p* < 0.001). At the same time, while compared with male individuals, female ones were characterized by a higher share of water (median of 25.81% vs. 19.4%; *p* < 0.001), and coffee/tea in their total daily beverage intake (median of 25.81% vs. 21.43%; *p* < 0.001).

The daily intake of beverages, in product sub-groups, assessed in a group of adolescents studied within the PLACE-19 Study, stratified by age, is presented in Table 5. When comparing beverages intake, there were no statistically significant age-dependent differences.

**Figure 2:**
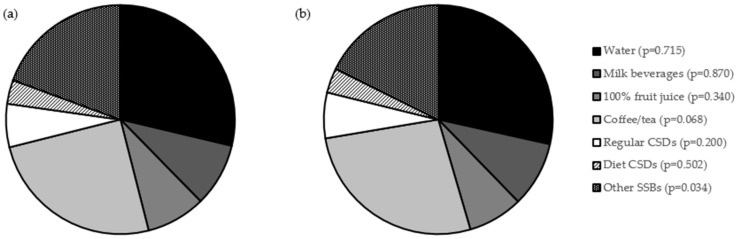
Share of daily intake of beverages, in product groups based on the survey questionnaire, assessed in a group of adolescents studied within the PLACE-19 Study (*n* = 984), stratified by age, for 15–17 years (**a**) and 18–20 years (**b**). Groups according to the National Youth Physical Activity and Nutrition Study (NYPANS) survey [27], based on the Nutrition Data System for Research (NSDR), beverage groups [32]; *p*-Values for Mann–Whitney U test; CSDs—carbonated soft drinks; and SSBs—sugar-sweetened beverages.

The share of daily intake of beverages, in product groups based on the survey questionnaire, assessed in a group of adolescents studied within the PLACE-19 Study, stratified by age, is presented in Figure 2. When compared with older individuals, younger ones were characterized by a higher share of other SSBs in their total daily beverage intake (median of 19.14% vs. 17.49%; *p* = 0.034).

The daily intake of beverages, in product sub-groups, assessed in a group of adolescents studied within the PLACE-19 Study, stratified by body mass, is presented in Table 6. When compared beverages intake, there were no statistically significant body mass-dependent differences.

**Figure 3:**
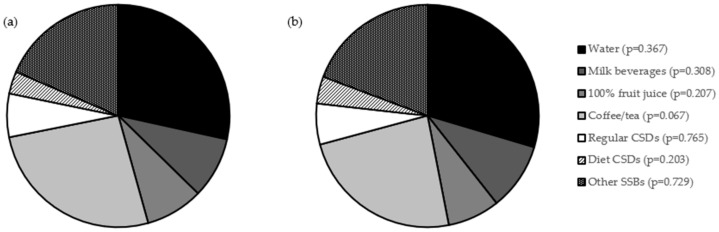
Share of daily intake of beverages, in product groups based on the survey questionnaire, assessed in a group of adolescents studied within the PLACE-19 Study (*n* = 984), stratified by body mass, for underweight/normal body weight (**a**) and excessive body mass (**b**). Groups according to National Youth Physical Activity and Nutrition Study (NYPANS) survey [27], based on the Nutrition Data System for Research (NSDR), beverage groups [32]; *p*-Values for Mann–Whitney U test; CSDs—carbonated soft drinks; and SSBs—sugar-sweetened beverages.

The share of daily intake of beverages, in product groups based on the survey questionnaire, assessed in a group of adolescents studied within the PLACE-19 Study, stratified by body mass, is presented in Figure 3. Similarly as for beverages intake, for the share of daily intake, there were no statistically significant body mass-dependent differences.

The total daily intake of beverages, compared with 80% of the AI levels, assessed in a group of adolescents studied within the PLACE-19 Study, is presented in Table 7. For the vast majority of the studied group, independently from the applied AI level, and a sub-group, the inadequate intake of beverages was observed, as over 70% of the studied group or sub-groups did not meet the 80% of the AI value. When comparing total daily intake of beverages between sub-groups, only for AI by IOM was there a gender-dependent difference, where a higher share of female individuals met the recommended value (15.7% vs. 8.1%, *p* = 0.0016).

## 4. Discussion

While assessing the obtained results, it seems that there are two problems related to beverage consumption—inadequate intake and inappropriate choices. In spite of the fact that analysis of the literature may have suggested it [16,17,18], the scale of the problem is quite serious, as over 70% of the studied population was characterized by inadequate beverage intake, and while combining the SSBs into one common group, for this specific group the highest intake was observed.

When comparing this observation with the results of the previous study by Kostecka et al. [17], presenting mean beverage consumption in Polish adolescents, it may be stated that the beverage consumption may be similar, as about 80% did not met daily beverage intake requirements in the referred study. The mean beverage consumption in the study by Kostecka et al. [17] was a little bit higher than in the HELENA-CSS [16], conducted in a population of adolescents from eight countries, which may confirm that while compared with the other European countries, not that the amount of consumed beverages but the choice may be most important issue for Polish population of adolescents. Especially very high intake of total SSBs, attributed to over 32% of total daily beverage consumption may be a worrying trend, when comparing it with the results of the analysis of the database of SSBs intake for children and adolescents aged 3–19 years from 185 countries with the result of 23% [19].

The high intake of SSBs in a population of adolescents is commonly associated with an inadequate knowledge about health risks related to consumption of this group of beverages [44]. Excessive intake of CSDs is linked to adverse health outcomes, including a higher risk of obesity, type 2 diabetes, cardiovascular diseases, hypertension, and reduced bone mineral density [45]. Moreover, an umbrella review of meta-analyses of observational studies by Lane et al. [46] identified an association between a higher SSBs consumption and a higher risks of depression, dental caries, and cardiometabolic diseases.

Importantly, even if adolescents know that SSBs are not within the health-promoting products, they often do not understand specific health risks, or in spite of the risks, they believe that SSBs may be consumed, or they even trust in the benefits of such products, where they indicate them as products that provide energy or replace electrolytes [47]. It corresponds with the results of the recent Polish study, conducted in a younger group, aged 11–13 years, as it was indicated that the share of children guided by advertising in their choice of beverages increased from 52.1% in 2018 to 58.5% in 2023 [17]. Considering this fact, nutritional education is necessary and should be focused on specific evidence-based information, including the clarification of common misinformation. Adolescents are vulnerable to misinformation and its influence is different than in the case of adults, as it is exerted through social influence, emotional manipulation and cognitive biases, where social media is a platform of broadening such misinformation and promoting specific nutritional behaviors [48].

It must be indicated that SSBs consumption is an element of broad lifestyle, and specific consumption model, as it is often associated with frequent choosing meals at fast-food restaurants and watching television for a long time [49]. Moreover, such model of behaviors is common for adolescents and their friends, as for SSBs consumption, and consuming meals at fast-food restaurants, the friends behaviors are associated [50]. In this age group, in spite of the fact that the parental attitude is not associated with intake of SSBs in their progeny, the parental intake of SSBs is associated with the intake in their progeny [51]. Taking this into account, it may be indicated that adolescents are prone to mimic nutritional behaviors of both their peers and parents, which is associated with a consumption of SSBs. However, it was observed that adolescents may change their behaviors both for the worse and for the better under the influence of their friends, while verbal encouragement alone is less effective [52]. Considering this fact, nutritional education should focus on knowledge as well as behavior modification and should include both adolescents, preferably in school settings, and their parents in the home environment.

Except for the high intake of SSBs, the low intake of water in the presented study may be indicated, where it corresponded an inadequate total beverage intake. It is also in agreement with the results of the study by Guelinckx et al. [18], as compared with the intake by children and adolescents from other countries (13 countries included to the study), the lowest intake of water was observed for Poland.

As indicated by Kozioł-Kozakowska et al. [53], for Poland in 2014, about 98% of the population had access to water from municipal water supplies with an adequate quality in accordance with the requirements on potable water quality, but over 60% of Polish people do not trust the municipal water supplies quality and are afraid to consume it without boiling. Similarly, at schools, children and adolescents may rather choose bottled water from school shops or vending machines [54]. Moreover, it is commonly indicated by Polish authors, that adolescents do not have free access to water at school, as well as do not have opportunity to drink water in the classroom, or even if they have it, they do not feel comfortable doing so [12].

However, a relatively high consumption of hot beverages was observed in the referred study by Guelinckx et al. [18], compared with the other countries, which may be attributed to tea consumption, and which was confirmed within the presented study. In order to discuss this result, it must be indicated that within some studies no significant differences between tea and water for hydration were observed [55]. Moreover, some authors observed no evidence of its diuretic action and additionally reported a positive effect on mood [56]. As a result, it may be stated that a high consumption of tea may potentially to some extent counteract the negative effect of low water intake.

In general, determinants of water consumption in adolescents are divided into four groups: individual (e.g., physiological and psychological variables), social (e.g., peer influence), environmental (e.g., availability and weather), and associated with policy (e.g., at school), but the majority of studies focus on individual determinants, and not on the other groups [57]. As a result, the role of social and environmental factors must be emphasized, and dedicated promotional campaigns are necessary to change the image of water. In addition to informative messages about the health benefits of drinking water, celebrities or athletes who appear active and energized while drinking water should be engaged [58]. As an example of such action, it is commonly indicated that while Cristiano Ronaldo, a Portuguese world-class football star, at a press conference for the Union of European Football Associations (UEFA) European Football Championships, in 2021, publicly rejected the product of the Coca-Cola company, being an official event sponsor, and indicated water, as preferred by him, the Coca-Cola company recorded a drop of $4 billion in the company market value [59]. This event took place in June 2021, while the presented study was conducted from May to June 2021, so the impact of this event was not observed within the studied group. However, this action by Cristiano Ronaldo is indicated as a turning point for world sport associations and sports competitions to stop promoting SSBs, and instead to join a global campaign to change beverage consumption patterns in a society [60].

Both traditional and digital marketing channels significantly influence SSB consumption patterns among younger age groups. While traditional methods like television and outdoor advertising remain impactful [61], digital strategies, including social media and influencer marketing also play an important role by increasing exposure and brand recognition, which may lead to higher consumption. Such association was observed within the study by Mustakim et al. [62] that indicated that viewing beverages advertised on social media is significantly related to SSB consumption behavior among urban students in Jakarta.

In spite of the fact that the conducted study allowed the formulation of novel observations for the Polish population, some limitations of the study must be indicated. First, due to its cross-sectional nature, this study cannot establish causality between the observed factors. At the same time, the assessment of the beverage consumption was based on the questionnaire data, which always is associated with the risk of self-reported bias, and it may have caused either underestimation of some beverages or the overestimation of the other ones. Moreover, the applied NYPANS survey questionnaire was adjusted for a Polish population, which may influence the measurement validity. Finally, these findings highlight the need for longitudinal studies to better understand the determinants of beverage intake.

## 5. Conclusions

The inadequate beverage intake in a population of adolescents may be a problem, especially considering the high intake of SSBs and relatively low intake of water. Especially in male, and younger individuals, due to a higher intake of SSBs than for female and older individuals, education is necessary to promote adequate intake and choices of beverages. Further research is needed in the area of beverage consumption to understand the determinants of beverage consumption and develop opportunities to improve it.

## Figures and Tables

**Table 1:** General characteristics of the group of adolescents studied within the PLACE-19 Study (*n* = 984).

Characteristics	Total (*n* = 984)		Female (*n* = 674)		Male (*n* = 310)	
	Mean ± SD	Median * (IQR)	Mean ± SD	Median * (IQR)	Mean ± SD	Median * (IQR)
Age (years)	16.8 ± 1.1	17.0 (2.0)	16.9 ± 1.1	17.0 (2.0)	16.8 ± 1.2	17.0 (2.0)
BMI (kg/m^2^)	21.8 ± 3.9	21.1 (4.9)	21.6 ± 4.0	20.7 (4.6)	22.4 ± 3.7	22.1 (4.9)
BMI percentile (*n* = 674) **	55.2 ± 31.0	58.0 (58.0)	53.0 ± 31.2	53.0 (57.0)	60.0 ± 30.0	65.0 (54.0)
	*n* (%)					
Body mass	Total (*n* = 984)		Female (*n* = 674)		Male (*n* = 310)	
Underweight	68 (6.9%)		54 (8.0%)		14 (4.5%)	
Normal body weight	697 (70.8%)		479 (71.1%)		218 (70.3%)	
Overweight	147 (14.9%)		89 (13.2%)		58 (18.7%)	
Obesity	72 (7.3%)		52 (7.7%)		20 (6.5%)	

* Non-normal distribution (Shapiro–Wilk test; *p* < 0.05); ** for individuals aged <18 years; Body Mass Index (BMI); SD—standard deviation; and IQR—Interquartile range.

**Table 2:** Daily intake of beverages, in product sub-groups, assessed in a group of adolescents studied within the PLACE-19 Study (*n* = 984).

Beverage Subgroups		Servings (250 mL) Per Day		Amount (mL) Per Day					
		Mean ± SD	Median * (IQR)	Mean ± SD	Median * (IQR)	Q1	Q2	Q3	Q4
Water		1.44 ± 1.4	1.00 (1.1)	360.40 ± 345.7	250.0 (285.0)	142.5	250.0	427.5	1428.0
Milk		0.40 ± 0.5	0.29 (0.5)	98.78 ± 133.8	72.5 (125.0)	17.5	72.5	142.5	1428.0
100% fruit juices		0.39 ± 0.4	0.29 (0.4)	97.71 ± 104.1	72.5 (107.5)	35.0	72.5	142.5	1073.0
Regular CSDs	Cola	0.20 ± 0.4	0.14 (0.3)	50.51 ± 86.8	35.0 (72.5)	0.0	35.0	72.5	1073.0
	Non-cola	0.12 ± 0.2	0 (0.1)	28.93 ± 59.6	0 (35.0)	0.0	0.0	35.0	715.0
Diet CSDs	Cola	0.11 ± 0.2	0 (0.1)	27.89 ± 51.9	0 (35.0)	0.0	0.0	35.0	427.5
	Non-cola	0.06 ± 0.2	0 (0)	15.01 ± 38.9	0 (0)	0.0	0.0	0.0	357.5
Other SSBs	Fruit drinks (different than 100% juices)	0.39 ± 0.5	0.29 (0.4)	96.69 ± 120.4	72.5 (107.5)	35.0	72.5	142.5	1428.0
	Flavored milk	0.07 ± 0.2	0 (0)	18.27 ± 49.5	0 (0)	0.0	0.0	0.0	715.0
	Bottled tea-based and coffee-based beverages	0.24 ± 0.4	0.14 (0.3)	58.64 ± 90.0	35.0 (72.5)	0.0	35.0	72.5	857.5
	Lemonade/flavored water	0.25 ± 0.4	0.14 (0.3)	61.78 ± 90.0	35.0 (72.5)	0.0	35.0	72.5	857.5
Isotonic drinks		0.12 ± 0.3	0 (0.1)	29.75 ± 76.2	0 (35.0)	0.0	0.0	35.0	1000.0
Energy drinks		0.20 ± 0.4	0 (0.3)	49.63 ± 93.0	0 (72.5)	0.0	0.0	72.5	1073.0
Fresh tea and coffee beverages	Coffee beverages	0.36 ± 0.5	0.14 (0.6)	90.16 ± 124.7	35.0 (142.5)	0.0	35.0	142.5	1073.0
	Tea beverages	0.71 ± 0.8	0.43 (0.9)	178.10 ± 196.4	107.5 (215.0)	35.0	107.5	250.0	1428.0
Total SSBs		1.58 ± 1.5	1.14 (1.6)	394.40 ± 375.1	285.0 (392.5)	142.5	285.0	535.0	3035.0

* Non-normal distribution (Shapiro–Wilk test; *p* < 0.05); SD—Standard Deviation; IQR—Interquartile range; CSDs—carbonated soft drinks; and SSBs—sugar-sweetened beverages.

**Table 3:** Share of daily intake of beverages, in product sub-groups, assessed in a group of adolescents studied within the PLACE-19 Study (*n* = 984).

Beverage Subgroups		Share of Daily Intake (%)					
		Mean ± SD	Median * (IQR)	Q1	Q2	Q3	Q4
Water		28.63 ± 20.2	23.81 (25.8)	13.6	23.8	39.5	100.0
Milk		7.71 ± 8.1	6.45 (9.9)	1.2	6.5	11.1	68.3
100% fruit juices		8.20 ± 6.6	7.14 (7.5)	3.6	7.1	11.1	53.6
Regular CSDs	Cola	4.03 ± 5.3	2.78 (6.5)	0.0	2.8	6.5	48.8
	Non-cola	2.22 ± 3.8	0 (3.7)	0.0	0.0	3.7	44.0
Diet CSDs	Cola	2.27 ± 3.8	0 (3.7)	0.0	0.0	3.7	27.3
	Non-cola	1.13 ± 2.5	0 (0)	0.0	0.0	0.0	20.0
Other SSBs	Fruit drinks (different than 100% juices)	7.95 ± 7.4	6.67 (8.8)	2.6	6.7	11.4	66.7
	Flavored milk	1.34 ± 3.0	0 (0)	0.0	0.0	0.0	19.1
	Bottled tea-based and coffee-based beverages	4.71 ± 6.3	2.47 (7.3)	0.0	2.5	7.3	50.0
	Lemonade/flavored water	4.98 ± 6.2	3.45 (7.7)	0.0	3.5	7.7	47.1
Isotonic drinks		2.10 ± 4.3	0 (3.0)	0.0	0.0	3.0	45.5
Energy drinks		3.84 ± 6.0	0 (5.9)	0.0	0.0	5.9	50.0
Fresh tea and coffee beverages	Coffee beverages	7.12 ± 8.6	5.16 (11.1)	0.0	5.1	11.1	60.0
	Tea beverages	13.90 ± 11.8	11.45 (13.0)	5.9	11.5	18.9	100.0
Total SSBs		31.17 ± 19.4	32.47 (31.6)	15.7	32.5	46.7	89.7

* Non-normal distribution (Shapiro–Wilk test; *p* < 0.05); SD—Standard Deviation; IQR—Interquartile range; CSDs—carbonated soft drinks; and SSBs—sugar-sweetened beverages.

**Table 4:** Daily intake of beverages, in product sub-groups, assessed in a group of adolescents studied within the PLACE-19 Study (*n* = 984), stratified by gender.

Beverage Subgroups		Female (*n* = 674)		Male (*n* = 310)		*p* **
		Mean ± SD	Median * (IQR)	Mean ± SD	Median * (IQR)	
Water		373.50 ± 349.8	250.00 (357.1)	331.90 ± 335.3	250.00 (250.0)	0.251
Milk		89.21 ± 126.9	71.43 (107.1)	119.70 ± 145.6	71.43 (142.9)	0.003
100% fruit juices		95.83 ± 101.0	71.43 (107.2)	101.80 ± 110.5	71.43 (107.1)	1.000
Regular CSDs	Cola	39.77 ± 74.9	35.71 (35.7)	74.11 ± 104.5	35.71 (107.1)	<0.001
	Non-cola	21.94 ± 46.3	0 (35.7)	44.38 ± 79.2	35.71 (35.71)	<0.001
Diet CSDs	Cola	23.69 ± 45.0	0 (35.7)	37.21 ± 63.5	0 (35.7)	0.003
	Non-cola	12.19 ± 34.7	0 (0)	21.31 ± 46.2	0 (35.7)	0.002
Other SSBs	Fruit drinks (different than 100% juices)	88.60 ± 102.3	71.43 (71.4)	114.20 ± 151.3	71.43 (107.1)	0.286
	Flavored milk	13.72 ± 39.1	0 (0)	28.17 ± 65.6	0 (35.7)	<0.001
	Bottled tea-based and coffee-based beverages	54.66 ± 84.1	0 (71.4)	67.32 ± 101.2	35.71 (71.4)	0.087
	Lemonade/flavored water	55.43 ± 81.0	35.71 (71.4)	75.58 ± 105.7	35.71 (107.1)	0.002
Isotonic drinks		22.25 ± 71.0	0 (0)	46.20 ± 84.5	0 (71.4)	<0.001
Energy drinks		43.37 ± 87.3	0 (71.4)	63.42 ± 103.3	35.71 (71.4)	0.002
Fresh tea and coffee beverages	Coffee beverages	93.95 ± 127.5	35.71 (142.9)	81.97 ± 118.1	35.71 (107.1)	1.000
	Tea beverages	186.40 ± 196.7	142.90 (178.6)	160.00 ± 194.9	107.10 (178.6)	0.096
Total SSBs		339.70 ± 322.1	250.00 (392.9)	513.5 ± 448.1	392.90 (433.0)	<0.001

* Non-normal distribution (Shapiro–Wilk test; *p* < 0.05); ** Mann–Whitney U test with Bonferroni correction; SD—Standard Deviation; IQR—Interquartile range; CSDs—carbonated soft drinks; and SSBs—sugar-sweetened beverages.

**Table 5:** Daily intake of beverages, in product sub-groups, assessed in a group of adolescents studied within the PLACE-19 Study (*n* = 984), stratified by age.

Beverage Subgroups		15–17 Years (*n* = 674)		18–20 Years (*n* = 310)		*p* **
		Mean ± SD	Median * (IQR)	Mean ± SD	Median * (IQR)	
Water		359.80 ± 337.8	250.00 (312.5)	361.60 ± 362.6	250.00 (285.7)	1.000
Milk		96.30 ± 128.7	71.43 (107.1)	104.30 ± 144.3	71.43 (138.4)	1.000
100% fruit juices		99.02 ± 106.9	71.43 (107.1)	83.29 ± 87.6	71.43 (71.4)	0.167
Regular CSDs	Cola	48.20 ± 86.0	35.71 (71.4)	55.82 ± 88.3	35.71 (71.4)	1.000
	Non-cola	29.40 ± 60.5	0 (35.7)	28.17 ± 57.6	0 (35.7)	1.000
Diet CSDs	Cola	28.90 ± 52.5	0 (35.7)	25.92 ± 50.7	0 (35.7)	1.000
	Non-cola	16.00 ± 41.8	0 (0)	13.02 ± 31.7	0 (0)	1.000
Other SSBs	Fruit drinks (different than 100% juices)	102.80 ± 132.4	71.43 (107.1)	94.81 ± 97.7	71.43 (71.4)	1.000
	Flavored milk	18.15 ± 45.8	0 (0)	18.55 ± 56.7	0 (0)	1.000
	Bottled tea-based and coffee-based beverages	60.24 ± 91.1	35.71 (71.4)	55.18 ± 87.5	35.71 (71.4)	1.000
	Lemonade/flavored water	61.25 ± 84.5	35.71 (98.2)	62.90 ± 100.9	35.71 (71.4)	1.000
Isotonic drinks		29.83 ± 77.5	0 (35.7)	29.72 ± 73.5	0 (35.7)	1.000
Energy drinks		53.09 ± 100.8	0 (71.4)	42.28 ± 73.0	0 (71.4)	1.000
Fresh tea and coffee beverages	Coffee beverages	85.71 ± 122.7	35.71 (107.1)	99.88 ± 128.7	71.43 (142.9)	1.000
	Tea beverages	172.30 ± 187.1	107.10 (178.6)	190.70 ± 215.0	142.90 (214.3)	1.000
Total SSBs		402.90 ± 377.5	321.40 (428.6)	376.00 ± 369.7	285.70 (357.1)	1.000

* Non-normal distribution (Shapiro–Wilk test; *p* < 0.05); ** Mann–Whitney U test with Bonferroni correction; SD—Standard Deviation; IQR—Interquartile range; CSDs—carbonated soft drinks; and SSBs—sugar-sweetened beverages.

**Table 6:** Daily intake of beverages, in product sub-groups, assessed in a group of adolescents studied within the PLACE-19 Study (*n* = 984), stratified by body mass.

Beverage Subgroups		Underweight/Normal Body Weight (*n* = 765)		Excessive Body Mass (*n* = 219)		*p* **
		Mean ± SD	Median * (IQR)	Mean ± SD	Median * (IQR)	
Water		358.90 ± 351.1	250.0 (285.7)	365.50 ± 326.6	250.0 (303.6)	1.000
Milk		96.64 ± 133.8	71.43 (125.0)	106.40 ± 133.6	71.43 (116.1)	1.000
100% fruit juices		96.92 ± 123.7	71.43 (71.4)	91.32 ± 93.3	71.43 (71.4)	1.000
Regular CSDs	Cola	51.73 ± 90.9	35.71 (71.4)	46.60 ± 70.5	35.71 (71.4)	1.000
	Non-cola	29.47 ± 60.6	0.0 (35.7)	27.40 ± 55.8	0.0 (35.7)	1.000
Diet CSDs	Cola	26.52 ± 49.7	0.0 (35.7)	32.94 ± 59.0	0.0 (35.7)	1.000
	Non-cola	14.38 ± 38.3	0.0 (0.0)	17.45 ± 40.8	0.0 (0.0)	1.000
Other SSBs	Fruit drinks (different than 100% juices)	99.52 ± 106.9	71.43 (107.2)	95.73 ± 108.5	71.43 (107.2)	1.000
	Flavored milk	18.14 ± 49.9	0.0 (0.0)	18.75 ± 48.1	0.0 (0.0)	1.000
	Bottled tea-based and coffee-based beverages	60.07 ± 92.2	35.71 (71.4)	53.65 ± 81.7	0.0 (71.4)	1.000
	Lemonade/flavored water	63.44 ± 93.5	35.71 (71.4)	55.94 ± 76.3	35.71 (71.4)	1.000
Isotonic drinks		29.88 ± 80.4	0.0 (35.7)	29.52 ± 59.8	0.0 (35.7)	1.000
Energy drinks		45.75 ± 81.7	0.0 (71.4)	63.44 ± 123.9	35.71 (71.4)	1.000
Fresh tea and coffee beverages	Coffee beverages	89.94 ± 126.1	35.71 (142.9)	91.0 ± 119.8	35.71 (142.9)	1.000
	Tea beverages	183.0 ± 200.1	107.10 (214.3)	161.10 ± 182.2	107.10 (178.6)	1.000
Total SSBs		395.50 ± 377.5	285.70 (392.9)	391.0 ± 367.4	285.70 (428.6)	1.000

* non-normal distribution (Shapiro-Wilk test; *p* < 0.05); ** Mann–Whitney U test with Bonferroni correction; SD—Standard Deviation; IQR—Interquartile range; CSDs—carbonated soft drinks; and SSBs—sugar-sweetened beverages.

**Table 7:** Total daily intake of beverages, compared with 80% of the adequate intake (AI) levels, assessed in a group of adolescents studied within the PLACE-19 Study (*n* = 984).

		% of Group Characterized by Adequate Intake for Total Daily Intake of Beverages					
		80% of AI by EFSA *		80% of AI by IOM **		80% of AI by PZH-NIH ***	
		%	*p*	%	*p*	%	*p*
Total		23.3	-	13.2	-	23.3	-
Gender	Female	25.1	0.0586	15.7	0.0016	25.1	0.0586
	Male	19.4		8.1		19.4	
Age	15–17 years	22.6	0.4795	12.9	0.7797	22.6	0.4795
	18–20 years	24.8		13.9		24.8	
Body mass	Underweight/normal body weight	24.1	0.3215	14.1	0.1531	24.1	0.3215
	Excessive body mass	20.5		10.0		20.5	

*Aged ≥14 years: 2.0 L for female and 2.5 L for male individuals [13]; ** aged 14–18 years: 2.3 L for female and 3.3 L for male individuals, aged ≥19 years: 2.7 L for female and 3.7 L for male individuals [14]; *** aged 13–15 years: 1.95 L for female and 2.35 L for male individuals, aged ≥16 years: 2.0 L for female and 2.5 L for male individuals [15]; AI—adequate intake; EFSA—European Food Safety Authority; IOM—Institute of Medicine; and PZH-NIH—Polish National Institute of Public Health.

## Data Availability

Data will be provided on request.

## Abbreviations

The following abbreviations are used in this manuscript:

AI	Adequate Intake
BMI	Body Mass Index
CAWI	Computer-Assisted Web Interview
CSDs	Carbonated soft drinks
CVD	Cardiovascular Disease
EFSA	European Food Safety Authority
FAO	Food and Agriculture Organization of the United Nations
FFQs	Food Frequency Questionnaires
HELENA-CSS	Healthy Lifestyle in Europe by Nutrition in Adolescence Cross Sectional Study
IOM	Institute of Medicine
IQR	Interquartile range
NCDs	Non-Communicable Diseases
NSDR	Nutrition Data System for Research
NYPANS	National Youth Physical Activity and Nutrition Study
PLACE-19	Polish Adolescents’ COVID-19 Experience Study
PZH-NIH	Polish National Institute of Public Health
SD	Standard Deviation
SSBs	Sugar-sweetened beverages
T2DM	Type 2 Diabetes Mellitus
UEFA	Union of European Football Associations
WHO	World Health Organization
